# Characterization of Potential Intoxications with Medicines in a Regional Setting

**DOI:** 10.3390/ph16020308

**Published:** 2023-02-16

**Authors:** Tânia Nascimento, Teresa Santos, Fátima Rato, Ana Luísa De Sousa-Coelho

**Affiliations:** 1 Escola Superior de Saúde, Universidade do Algarve (ESSUAlg), Universidade do Algarve, Campus de Gambelas, Edifício 2, 8005-139 Faro, Portugal; 2 Algarve Biomedical Center Research Institute (ABC-RI), Universidade do Algarve, Campus de Gambelas, Edifício 2, 8005-139 Faro, Portugal; 3 Faculdade de Ciências e Tecnologia, Universidade do Algarve (FCT-UAlg), Universidade do Algarve, Campus de Gambelas, Edifício 2, 8005-139 Faro, Portugal; 4 Centro de Informação Antivenenos (CIAV), 1000-113 Lisboa, Portugal; 5 Instituto Nacional de Emergência Médica (INEM), 1000-113 Lisboa, Portugal; 6 Algarve Biomedical Center (ABC), Universidade do Algarve, Campus de Gambelas, Edifício 2, 8005-139 Faro, Portugal

**Keywords:** Algarve, drug intoxication, Portuguese Poison Information Center, toxicology

## Abstract

The Portuguese Poison Information Center (from Portuguese—CIAV) is a call center that offers medical assistance in case of possible intoxication with any kind of product, including medicines. This center´s main goal is to inform and guide the general public and health professionals. This work aimed to analyze and compare data corresponding to the telephone calls from the Algarve region (South of Portugal), received by CIAV during 2019 and 2020, regarding potential intoxications with medicines. To this end, data provided by CIAV on possible cases of medication intoxication in the Algarve region were collected, including the number of calls received, the place of origin of the call, the age group and sex of the intoxicated individual, the route of exposure to the drug, the circumstances of contact with the substance, the existence of symptoms, and the drug or drugs involved in the potential intoxication. The results showed that the number of cases slightly decreased in 2020 (*n* = 1261) compared with 2019 (*n* = 1340), with a high number of cases of intoxication in children between one and four years old in both years (21.2%; *n* = 152 in 2019; 16.4%; *n* = 115 in 2020). The drugs belonging to the locomotor system group (paracetamol and ibuprofen) were the main drugs involved, followed by the central nervous system pharmacotherapeutic group, namely benzodiazepines (diazepam and alprazolam). Paracetamol was the main drug responsible for the calls to CIAV (*n* = 71 in 2019; *n* = 63 in 2020), while for the remaining drugs there were fluctuations in their positions between both years. In some cases, this swinging may be explained by the possible changes in therapy due to potential interactions with drugs used for the treatment of symptoms of COVID-19 or perhaps related to misleading information released by the media about the use of some drugs, such as ibuprofen, during lockdown periods. Although there has been a decrease in calls to report possible drug intoxication in the Algarve region, the profile of calls was very similar. Paracetamol was the drug with the highest number of reported cases and the group of psychotropic drugs showed the largest increase between 2019 and 2020.

## 1. Introduction

The detection of a case of intoxication requires specialized medical personnel who can identify the characteristic symptoms of the episode. In some cases, the symptoms of drug intoxication are not specific and can even be masked by other conditions making it difficult to detect the intoxication and the toxic agent [1,2]. Before diagnosing the cause of intoxication, health care professionals should assess the patient’s vital signs and determine whether or not they need urgent treatment. Most intoxications occur at home, with medicines being the main toxic agents involved in these conditions, which contact is mostly made by ingestion [3,4,5]. Drug intoxications are important and often understudied, although they are considered a public health problem. The number of deaths from drug intoxication has been increasing. It is estimated that over 8000 overdose deaths occurred in the European Union in 2017 [6]. However, most of the published results on drug intoxications refer to illicit drugs, being scarce in Europe and especially insufficient from Portuguese data. 

Established in 1982, the Portuguese Poison Information Centre (CIAV, from Portuguese *Centro de Informação Antivenenos*), is the only toxicology center in Portugal that collects data and information on intoxications in the country. The CIAV issues an annual report on intoxications registered in the Portuguese Poison Centre where data on drug intoxications can be found [7]. Data from the 2021 report showed that in Portugal, the anxiolytic/antidepressant/antipsychotic group was the most involved in intoxications (37.3%), followed by the analgesic/anti-pyretic/anti-inflammatory group (10.15%) [8]. Similar data were obtained in a study carried out in China, where acute intoxications were mainly caused by sleeping pills (24.22%), analgesics (20.31%), and antipsychotics (16.41%) [9]. In Europe, as in the United States of America, the substances most associated with overdoses include analgesics drugs [8,10]. America’s Poison Centers report shows that in the last 10 years there has also been an increase in intoxications related to antidepressants (5.3%/year) [10]. However, data on fatal drug intoxication in Spain between 2000 and 2018 showed that in 2018, 72.6% of these intoxications were accidental and due to non-psychotropic/non-specified drugs. Although intoxications from psychotropic drugs were prevalent until 2004, from that date onwards the prevalence started to decrease. Anticoagulant drugs (43.5%) and cardiac-stimulant glycosides (26.0%) were the drugs most involved in fatal intoxications [11]. Therefore, it is important to know the profile of intoxication caused by medications so that preventive action can be taken, particularly at the level of health care providers directly related to medicines, such as physicians, pharmacists, or nurses [12].

The aim of this work was to analyze the data provided by CIAV regarding calls due to potential drug intoxication that occurred in the Algarve region (Portugal) during the years 2019 and 2020. This analysis also aims to describe and characterize the cases occurring in a period marked by the onset of the global pandemic of COVID-19 [13].

## 2. Results

### 2.1. Calls Received by CIAV: Number, Type of Consultant and Call Origin

CIAV provides telephone assistance to health professionals or the general public in cases of intoxication and information on the toxicology of a certain drug. In 2019, a total of 26,955 calls were received, of which 1340 were from the district of Faro (Algarve), where 58% (*n* = 773) were related to potential drug intoxications. In 2020, the total number of calls was 22,531, corresponding to 1261 calls made by the district of Faro, where 57% (*n* = 714) were related to drug intoxications. The remaining calls were related to requests for information or to intoxications by other non-medicinal substances.

**Figure 1:**
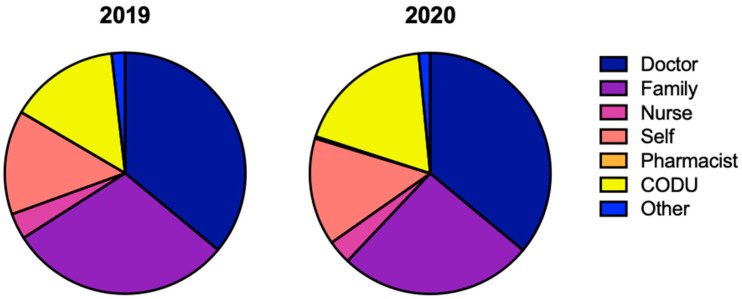
Characterization of the contact person to the Poison Information Centre in the Algarve region in 2019 and 2020. Comparing 2019 and 2020, the distributions were as follows: doctor (35.96% vs. 35.99%), family (30.01% vs. 26.05%), nurse (3.49% vs. 3.22%), self (13.97% vs. 14.43%), pharmacist (0% vs. 0.28%), CODU (14.75% vs. 18.49%), other (1.81% vs. 1.54%). Abbreviations: CODU—Urgent Patient Orientation Centre, from the Portuguese *Centro de Orientação de Doentes Urgentes*.

In the Algarve region, around 36% of the calls were made by a medical doctor in both periods (35.9%, *n* = 278 in 2019; 35.9%, *n* = 257 in 2020), followed by the family member of the potentially intoxicated individual (30.0%; *n* = 232 in 2019 and 26.1%; *n* = 186 in 2020) (Figure 1). While in 2019 there were no calls made by pharmacists, in 2020 there were two calls by pharmacists. In 2020, there was a 4% decrease in calls made by family members and a 4% increase in calls made by operatives from the Urgent Patient Orientation Center (CODU, from Portuguese *Centro de Orientação de Doentes Urgentes*), compared with 2019. Regarding the remaining callers, there was no considerable fluctuation (Figure 1).

**Figure 2:**
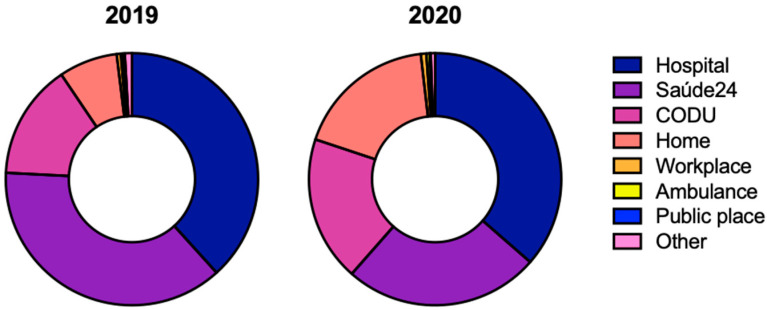
Characterization of the place of origin of the call to the Poison Information Centre in the Algarve region in 2019 and 2020. Comparing 2019 and 2020, the distributions were as follows: hospital (38.29% vs. 36.41%), saúde24 (37.52% vs. 25.07%), CODU (14.75% vs. 18.63%), home (7.50% vs. 18.07%), workplace (0.52% vs. 0.70%), ambulance (0.26% vs. 0.42%), public place (0.26% vs. 0.14%), other (0.91% vs. 0.56%). Saúde24 (SNS24) is a healthcare hotline, i.e., a telephone and online service of the Portuguese National Health Service (SNS). Abbreviations: CODU—Urgent Patient Orientation Centre, from the Portuguese *Centro de Orientação de Doentes Urgentes*.

The origin of the call made to CIAV may or may not be the same location where the individual was originally exposed to the product. There was a predominance of the hospital/health center as the place of origin of the call, both in 2019 (38.3%; *n* = 296) and in 2020 (36.4%; *n* = 260) (Figure 2). While calls from the home increased, calls from the national healthcare hotline (Saúde24) decreased in 2020 (Figure 2). A public place is the location with the lowest number of calls made in both 2019 (0.3%; *n* = 2) and 2020 (0.1%; *n* = 1) (Figure 2).

### 2.2. Characterisation of Intoxications

**Figure 3:**
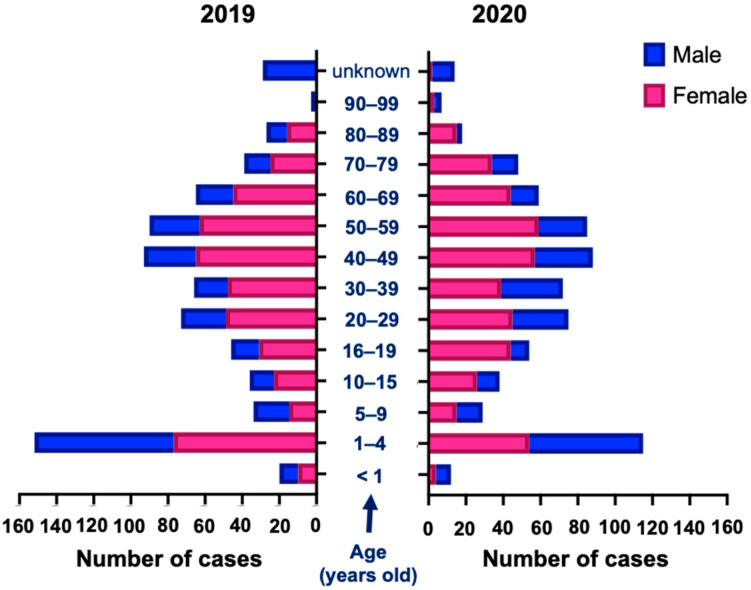
Distribution of cases by age group and sex during 2019 and 2020. Comparing 2019 and 2020, the distributions were as follows by age group and sex: <1 years old (2.14% F; 3.28% M vs. 0.91% F; 2.93% M), 1–4 (16.45% F; 24.59% M vs. 12.24% F; 22.34% M), 5–9 (3.21%; 6.23% M vs. 3.40% F; 5.13% M), 10–15 (4.91%; 4.26% M vs. 5.90% F; 4.40% M), 16–19 (6.62%; 4.92% M vs. 9.98% F; 3.66% M), 20–29 (10.47%; 7.87% M vs. 10.20% F; 10.99% M), 30–39 (10.26%; 5.90% M vs. 8.84% F; 12.09% M), 40–49 (13.89%; 9.18% M vs. 12.93% F; 11.36% M), 50–59 (13.46%; 8.85% M vs. 13.38% F; 9.52% M), 60–69 (9.62%; 6.56% M vs. 9.98% F; 5.49% M), 70–79 (5.34%; 4.59% M vs. 7.71% F; 5.13% M), 80–89 (3.42%; 3.61% M vs. 3.40% F; 1.10% M), 90–99 (0.21%; 0.66% M vs. 0.68% F; 1.47% M), unknown age (0% F; 9.51% M vs. 0.45% F; 4.40% M). F, females; M, males.

Any individual is at risk of drug intoxication by administering a drug incorrectly or in an excessive dose. In both 2019 and 2020, the female gender represented about 63% (62.9%, *n* = 468 in 2019; 62.7%, *n* = 441 in 2020) of the cases. In both years, when analyzed by age groups (Figure 3), the female gender predominates from the age group of 10–15 years and older, while the male gender predominates in younger ages (Figure 3). In adults, the highest prevalence of potential intoxication cases corresponded to individuals aged between 40 and 59 years in both 2019 (24.6%; *n* = 183) and 2020 (24.7%; *n* = 173). In children, it was the age group between 1 and 4 years (21.2%; *n* = 152 (*n* = 77 female; *n* = 75 male) in 2019; 16.4%; *n* = 115 (*n* = 54 female; *n* = 61 male) in 2020) (Figure 3).

The route of exposure of most medicines administered in outpatient clinics is the oral route, thus being the route by which most intoxications occurred. In 2019, the most representative route of exposure corresponds to the digestive route (oral and rectal) with 737 cases (94.4%), followed by the inhalation route with 14 cases (1.8%). In 2020, the data were similar, being the digestive route the most representative with 667 cases (92.8%) and the inhalation route the second most common route of exposure (1.25%; *n* = 9).

**Figure 4:**
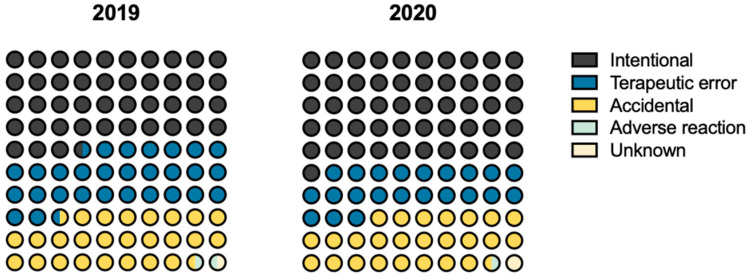
Characterization of the intoxication circumstances in the Algarve region in 2019 and 2020. Comparing 2019 and 2020, the distributions were as follows: intentional (43.47% vs. 51.19%), therapeutic error (29.11% vs. 21.74%), accidental (25.74% vs. 25.39%), adverse reaction (1.03% vs. 0.84%), unknown (0.65% vs. 0.84%).

The circumstance of intoxication may be one of the most important points to understand the reason and thus prevent future occurrences. The intentional circumstance may occur either by suicide intention or by conscious intake of the drug, by taking more than the maximum daily dose. Intentional exposure represented about 44% (*n* = 336) and 51% (*n* = 365) of the intoxications that occurred in 2019 and 2020, respectively (Figure 4). The following most common circumstances were identified as accidental (unintentional) (approximately 25% in both 2019 (*n* = 199) and 2020 (*n* = 181)) or therapeutic error, where the latest showed a decrease of 7% in 2020 (Figure 4).

**Figure 5:**
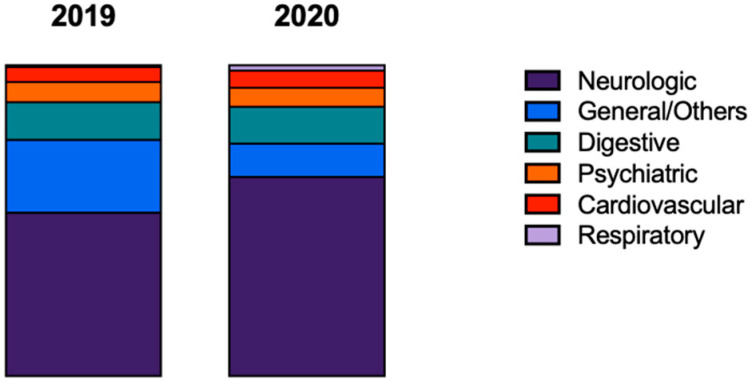
General symptoms grouped by main system affected described in the calls to the Poison Information Centre in the Algarve region in 2019 and 2020. Comparing 2019 and 2020, the distributions were as follows: neurologic (52.54% vs. 64.06%), general/others (23.45% vs. 10.73%), digestive (12.15% vs. 11.88%), psychiatric (6.45% vs. 6.09%), cardiovascular (4.80% vs. 5.51%), respiratory (0.57% vs. 1.74%). Percentages were calculated by the total of symptomatic cases.

Depending on the drug and route of exposure, the symptoms of intoxication may be different affecting different organs at different levels [14]. Approximately half of cases (54.5% (*n* = 458) and 47.7% (*n* = 330) in 2019 and 2020, respectively) were asymptomatic. When considering the symptoms identified (not shown), the symptoms at the neurological level (mainly drowsiness) were the most felt (showing an increase in 2020), followed by symptoms at the digestive level (Figure 5).

The CIAV physician who receives the calls, specialized in toxicology, is responsible for orienting the potentially intoxicated individual according to the to some parameters, such as the toxicity, the circumstances, and the individual. There were no differences between the guidance given to the individuals in the studied years. It was found that around 40% of the patients were advised to remain at home. On the other hand, 30% of the cases were under hospital surveillance for more than 24 h, meaning those calls were performed on behalf of recently hospitalized individuals. About 16% were advised to go to a hospital, having in mind the potential consequences of poisoning, meaning those individuals were at home or somewhere else but not at the hospital. The remaining kinds of orientation included medical consultation, internal transfers from medical services or discharge from hospital, or the orientation was unknown (i.e., not registered).

### 2.3. Pharmacotherapeutic Groups and Drugs Involved

**Figure 6:**
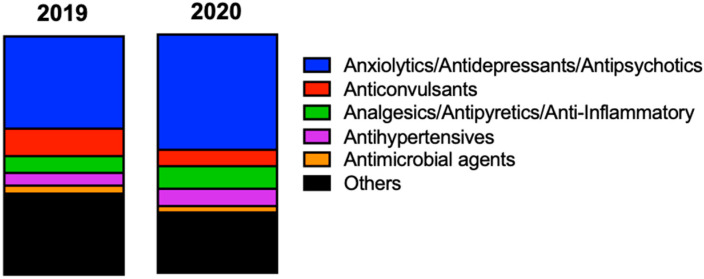
Characterization of the pharmacotherapeutic groups most involved in potential drug intoxications in the Algarve region in 2019 and 2020. Comparing 2019 and 2020, the distributions were as follows: anxiolytics/antidepressants/antipsychotics (38.74% vs. 48.40%), anticonvulsants (11.49% vs. 6.85%), analgesics/antipyretics/anti-inflammatory (7.04% vs. 9.50%), antihypertensives (5.38% vs. 7.29%), antimicrobial agents (3.34% vs. 2.65%), others (34.01% vs. 25.30%).

Both prescription and non-prescription (over-the-counter) medicines may be at the origin of intoxications; however, it is the prescribed medicines belonging to the central nervous system pharmacotherapeutic group that are mainly responsible for intoxication cases (Figure 6). Considering the pharmacotherapeutic group, it was found that both in 2019 (38.7%; *n* = 418) and 2020 (48.4%; *n* = 438), the group of psychotropic drugs was the most involved in potential intoxications, namely benzodiazepines (15.9%, *n* = 172 in 2019; 22.5%, *n* = 199 in 2020). Also, antipsychotics showed an increase in 2020 (12.3%; *n* = 109) compared with 2019 (8.25%; *n* = 89) (Table S1).

Regarding the group of antimicrobial agents, aminopenicillins were mainly responsible for the high number of intoxications in this group in 2019 (36.1%; *n* = 13). However, a decrease was observed in 2020 (10.5%; *n* = 2) (Table S1). Potential intoxication from analgesics, antipyretics, and nonsteroidal anti-inflammatory drugs accounted for about 13% of potential drug intoxication (Table S1). In both 2019 (50.7%; *n* = 76) and 2020 (64.2%; *n* = 86), analgesics and antipyretics (including narcotic analgesics) were the groups with the highest proportion of potential intoxications (Table S1). Regarding the group of the locomotor system, the nonsteroidal anti-inflammatory drugs derived from propionic acid (ibuprofen and naproxen) were responsible for the largest number of intoxications in this group (Table S1).

Analyzing the active substances most involved in potential drug intoxication, we found paracetamol was the main cause of potential intoxication in both years (Table 1). In 2020, there was a decrease and an increase of five places for ibuprofen and diazepam, respectively, compared with 2019 (Table 1). In the case of ibuprofen, there was a decrease by almost half in the number of cases (3.16%; *n* = 28) compared with 2019 (5.24%; *n* = 59). In 2019, diazepam was the 7th substance responsible for medication intoxications, reaching 2nd place in 2020, with a doubling in the number of calls. Quetiapine was the third most important drug responsible for potential drug intoxications in the Algarve in 2020, with an increase of 21% compared with 2019. Alprazolam was 4th most responsible for intoxications occurring in both 2019 and 2020. Clonazepam was the 5th main drug responsible for the intoxications in 2020, showing a decreasing trend of 31% compared with 2019 (Table 1). 

## 3. Discussion

Although considered a public health problem, intoxications with medicines are often understudied. From the analysis of the data corresponding to the telephone calls received by the Portuguese Poison Information Center (CIAV) during 2019 and 2020, regarding potential intoxications with medicines, it was found that the district of Faro (the Algarve region) accounted for 5% of the nationwide calls to the CIAV. According to the National Institute of Statistics, the district of Faro represented 4.5% of the population of Portugal in 2021 [15], which corresponds to a population proportional to the number of calls from that district. Thus, it can be considered that Faro does not represent a district with an excess of potential intoxication cases. According to national level data, the number of calls in Portugal decreased by 9% in 2020 compared with 2019 [16], possibly related to the decrease in global activity due to the COVID-19 pandemic and the confinement periods [17]. The total number of calls has gradually declined from 2016 to 2021. Different reasons may be at the origin of this decrease in cases. On one hand, there might have been an evolution in the education of the population over the years regarding the conscious administration of medication and the dangers it may represent. On the other hand, there is an increasing effort performed by pharmacists regarding the information provided in the dispensing of the medication [18]. Despite the overall decrease in the number of calls, the proportion of calls regarding potential intoxication by medication remained close to 60% in both years [19].

The patient, when experiencing symptoms of intoxication or when becoming aware that a higher than maximum recommended dose was administered, either contacts the emergency medical number or goes to the hospital/health center, justifying the results obtained. Between 2019 and 2020, there was an increase in calls made from home, presumably due to the mandatory confinement imposed on all citizens by the Portuguese government due to the COVID-19 pandemic, which decreased the access of individuals to hospital services [20,21]. There was, however, a decrease in calls received from SNS24. The *Serviço Nacional de Saúde 24* (SNS24 or Saúde24) is a 24 h call center of the National Health Service available throughout the year, which provides citizens with therapeutic advice on issues related to illnesses and medications [22]. The overload of SNS24 telephone lines, which were specifically set up to counsel possible cases of COVID-19 in 2020, may be at the origin of this decrease. At the national level, in 2019 most calls to the CIAV were made from the SNS24, followed by the CODU, hospital/health center, and in a smaller number, from home or public places [16]. Already in the year 2020, these data changed from the previous year since most of the calls were made from home or a public place and the minority from the workplace and SNS24, suggesting that the pandemic has changed the way drug intoxications were approached by both the general public and health professionals [23,24]. The data analyzed only referred to the call and not to the location where the intoxication occurred. In the event of an intoxication, probably due to not being aware about the existence and functioning of the CIAV, most citizens in Portugal when victims of intoxication will call the SNS24 or to the CODU instead of calling directly to the CIAV.

Regarding the potentially intoxicated individual, in both 2019 and 2020, females accounted for the majority of cases (63%). Indeed, it was described females seek medical treatment more often than males [25,26], and for this reason they may be more likely to suffer from drug intoxication. 

Regarding the age distribution, 40 to 59 years old adults showed a high prevalence of intoxications, representing almost 25% in both years. This age group corresponds to 29.7% of the population in Portugal [27] reflecting a relative proportion, which may explain why this group reaches a high number of intoxications in both years. According to a study conducted between 2001 and 2013, in the forensic medicine services of northern Portugal it was found that 55% of adults aged between 40 and 59 years are chronic consumers of medicines [28]; therefore, there is a substantial risk of accidental medicament intoxication or their side effects. It is important to note that children aged 1 to 4 years showed a high incidence of potential drug intoxication, which partially decreased in 2020. Individuals belonging to this age group are at risk of accidentally ingesting medication that is within their reach and without adult supervision. On the other hand, they also run the risk of being given medication by their relatives in the wrong way, especially if it is through self-medication. An error may also occur in the dose administered or the route of administration. Adverse reactions can also be at the origin of these intoxications since in the first years of life there may still be no knowledge about the individual’s intolerances [29,30]. According to the data corresponding to the years 2016 to 2020 in Portugal, the number of cases of drug intoxication in the 1–4 age group also represents the highest number of cases at a national level [16]. The period of confinement and the COVID-19 pandemic apparently may have influenced the potential intoxications that occurred in the year 2020 in this age group in the Algarve region compared with the previous year, as there was a decrease in the number of cases. The increase in the amount of medication at home, especially painkillers prescribed to adults, and the fact that formulation of children’s medications is designed to be sweetened might be conceivable reasons to induce children to take them unsupervised [31].

The circumstances of drug intoxication may or may not contribute to understand the causes responsible for intoxications. The most common cause is intentional intoxication, which accounts for almost half of the cases in both 2019 and 2020, with an increase of 8% in 2020 compared with 2019. Intentional intoxication may or may not correspond to suicide cases; however, there are no data in this study to inform how many suicide cases occurred in the two years under study. However, the results of a study conducted in Barcelona indicate that there was an increase of 43.2% in suicide thoughts and attempts during the pandemic compared with the 2018–2019 period, reaching a maximum increase of 573.8% in people under 18 years of age in the month of May 2021 [32]. Also, the “Report of specific developed activities of CODUs” for 2020 shows that suicidal behaviors increased in the second half of 2020, with an expected trend of gradual increase according to the phases of crisis response mechanisms [16]. From the data it can be inferred that part of the increase in cases due to intentional circumstance can probably be linked to suicide. Not surprisingly, the main route of administration of the medicines causing intoxication was the oral route. The fact that most medicines in outpatient settings are administered orally [33] may explain these data.

The two-year period in the analysis includes an atypical year where a containment and consequent decrease in cases of influenza was observed in 2020 [34]. This may have contributed to a decrease in the use of medication for this pathology and other communicable respiratory diseases and, consequently, may contribute to the decrease in number of cases due to therapeutic error. As seen in the Algarve region in Portugal, most cases were intentional intoxication. Compared with the average of cases in previous years and in 2020 there was, as in the Algarve region, a decrease of about 8% of intentional intoxications. The second most common cause is accidental, with an increase of 3% in 2020 compared with the average of cases in previous years, which may have resulted from the decrease in the medical surveillance of patients affected by the COVID-19 pandemic. Intoxication caused by therapeutic error decreased nationally by about 22% in 2020 compared with the average of previous years [16], a more significant decrease compared with the Algarve.

In half of the cases observed in this study, the patients did not present any symptoms. This proportion partially fits with the most common orientation given, being to stay at home (40%). This also may indicate that the call to CIAV was made soon after the administration of the drug, allowing advice on immediate measures to avoid more serious consequences. The main symptoms that occurred were at the level of the neurological system, possibly because most intoxications occurred due to the consumption of medicines belonging to the central nervous system group, followed by symptoms at the level of the digestive system related to the main route of administration, oral ingestion. However, the data available for this study do not determine whether the overconsumption of medicines occurred in each case of intoxication. 

As in this study, psychotropic drugs are one of the most reported examples of potential drug intoxication [35]. According to the National Authority for Medicines and Health Products—Infarmed (in Portugal), there has been an increase in the consumption of psychotropic drugs in Portugal [36]. Psychiatric disorders have a prevalence of 22.9% in Portugal and depression affects 10% of the population [37]. Despite their beneficial properties to a wide number of diseases, these drugs pose certain risks and may lead to habituation and dependence. The significant increase in the use of antidepressants reflects the preferential use of pharmacological treatment and the prevalence of mental disorders in Portugal [38]. Also, a retrospective study conducted in the Intensive Care Medicine Service of the Coimbra University Hospitals (Center of Portugal) between the years 2002 and 2014 showed a high prevalence of intentional intoxications (88.9%), namely due to medicines (35.0%) [39]. Among the intoxications due to medication, those due to the consumption of benzodiazepines (50.4%), antidepressants (46.4%), or other psychotropic drugs (33.0%) stood out during the 11 years of the study. This work also showed that in the study period there was an increase in the proportion of drug intoxications [39].

The general confinement of the population, the use of mouth masks, the frequent washing and disinfection of hands and surfaces, and the social distancing contributes to reduce the risk of transmission of both viral and bacterial infections [40], and as a result, reduces the need for antibacterial agents. According to the 2021 annual report of the Directorate General of Health (DGS, from Portuguese *Direção Geral da Saúde*) on the national program for infection control and antimicrobial agent resistance, in 2020 there was a decrease in the consumption of antimicrobial agent consumption in the outpatient settings with a decrease of 23% compared with 2019. Nonetheless, the number of cases of drug intoxication by antimicrobial agents decreased by about 51% in 2020 compared with 2019, i.e., the decrease in cases of intoxication was approximately double the decrease in their consumption [41]. In the present work, the decrease in potential intoxication by antimicrobial agents showed a decrease of about 25%.

Most drugs belonging to the group of analgesics, antipyretics, and nonsteroidal anti-inflammatory drugs are, in Portugal, non-prescription medicines (i.e., over the counter (OTC)). According to Infarmed’s 2020 Annual Report on the Sales of Non-Prescription Medicines, there has been a slight increase in the sales of these medicines, with analgesics and antipyretics accounting for 25% of the total OTC sales [42]. In the Annual Report of the American Association of Poison Control Centers’ National Poison Data System (NPDS): 38th Annual Report (2020), it is also stated that the group of analgesics is the most prevalent in drug intoxications (10.5%) [24].

Portugal is one of the countries with the highest consumption of anxiolytics, hypnotics, and sedatives, including, mostly benzodiazepines and analogues [43]. These drugs were mainly responsible for the potential drug intoxications that occurred in 2019 and 2020. Regarding the central nervous system group, benzodiazepines are mainly responsible for intoxications with an increase of 15% of cases in 2020 compared with 2019, only in the Algarve region. Zolpidem, a non-benzodiazepine receptor modulator used in the short-term treatment of insomnia, which improves measures of sleep latency, sleep duration, and reduces the number of awakenings in patients with transient insomnia [44], showed a 76% decreasing trend in 2020 from the previous year. According to Liu K. *et al*., there may be reasons for the increased consumption of some hypnotics and decreased consumption of others. Due to the COVID-19 pandemic, many individuals developed sleep disorders, a consequence of isolation or medication use and, therefore, the prescription of hypnotic medications was necessary to support sleep [45]. Other individuals may have had the need to alter their hypnotic therapy arising from COVID-19 infection. Major factors that may influence the prescription of hypnotic medications in sick individuals with COVID-19 include respiratory depression caused by hypnotic medications, interactions between hypnotics and medications used to treat COVID-19, and changes in hypnotic drug metabolism due to liver damage caused by COVID-19 drug therapy [46]. The risk of respiratory infection after zolpidem consumption and the risk of influenza viral infection after zolpidem have also been reported. In fact, among hypnotic drugs, most respiratory complications are observed with zolpidem [45].

Ibuprofen, which in 2019 ranked 2nd as the drug substance responsible for intoxication, moved to 7th place in 2020. In that year, the French authorities warned against the use of ibuprofen in patients infected with COVID-19, arguing that it worsened the symptomatology of the infection [47], information that was later contradicted [48,49,50]. Media reports may justify this decrease, as may the decrease in exposure and transmission of the general population to agents causing infection, which require common anti-inflammatory drugs. Comparing the 10 most consumed medicines in outpatient settings in Portugal in 2019 and 2020, according to information issued by Infarmed [51] with the highest-ranked medicines involved in drug intoxications (from this work), only paracetamol and alprazolam are part of both ranks, thus demonstrating that there is no direct relationship between the consumption of certain active substances and potential intoxications.

## 4. Materials and Methods

A descriptive retrospective study analyzed data collected by CIAV. Of all national calls, only those made from the Algarve region in the period 1 January 2019 to 31 December 2020 were analyzed. The total number of calls, the age group and gender of the individual, the route of administration of the medication, the circumstances under which the intoxication occurred, the symptoms reported, and the advice that was provided were collected. 

The drugs involved in the intoxications were classified by the pharmacotherapeutic classification contained in the Online Pharmaceutical Record [52]. For the classification, only the active substance of the drug was considered, regardless of the pharmaceutical form, excipients, or dosage. Medicines with two or more active substances in their composition were counted separately by each of their active substances. The database provided by CIAV contained ethanol as a substance considered for the report of potential drug intoxication; however, given the objectives of the present study, this substance was not accounted for in the 10 highest-ranked medicines involved in potential cases of drug intoxication.

Symptoms experienced were grouped based on the system affected, namely neurologic (drowsiness, dizziness, headache, ataxia, coma, unconsciousness, tremors, and convulsions), digestive (nausea, abdominal pain, and vomits), psychiatric (behavioral changes), cardiovascular (tachycardia, hypotension, hypertension, and fibrillation), respiratory (dyspnea and pneumonia), and general/others (prostration and other).

Descriptive statistics were performed using SPSS v.28. 

## 5. Conclusions

We can, therefore, conclude that between 2019 and 2020, there was a decrease in calls of possible drug intoxication in the Algarve region. However, the profile of the consultant, the location of the call, the socio-demographic characteristics of the individuals who suffered the intoxication, the high number of cases of intoxication in children aged between 1 and 4 years, were all relatively similar. The pandemic situation and the confinement changed the routine of a large number of the citizens, also changing the habits of the consumption of medicines, resulting in a marked variation of the medicines responsible for drug intoxications. However, paracetamol remained the drug that caused the most cases of intoxication. 

Therefore, it is important to provide patients with information about medicines so that they can take them safely, maximizing the benefits and minimizing the risks associated with pharmacotherapy. Promoting health and safety education among citizens becomes very important for enhancing the value of the conscious use of medication. The intervention of health professionals is crucial through pharmacovigilance, the system responsible for monitoring the safety of active medicines on the market, which collects reports of adverse reactions in order to assess the risks associated with the use of medicines and implement measures to minimize them. Increasing awareness and education of the general population, especially in what concerns risks to children, and on how to use medicines correctly and safely through more direct interventions by health professionals will certainly lead, in the future, to fewer cases of intoxication in the population.

## Figures and Tables

**Table 1:** Drugs involved in intoxications grouped in descending order relative to the years 2019 and 2020, according to the number of cases identified.

*Rank*	*n*	2019	*n*	2020
*1*	71	Paracetamol	63	Paracetamol
*2*	59	**Ibuprofen**	55	**Diazepam**
*3*	42	Clonazepam	47	Quetiapine
*4*	40	Alprazolam	38	Alprazolam
*5*	39	Quetiapine	29	Clonazepam
*6*	26	Mexazolam	28	**Ibuprofen**
*7*	26	**Diazepam**	28	Bromazepam
*8*	25	Zolpidem	28	Lorazepam
*9*	23	Escitalopram	25	Sertraline
*10*	22	Lorazepam	23	Olanzapine

## Data Availability

Data is contained within the article and supplementary material.

## Supplementary Materials

The following supporting information can be downloaded at: https://www.mdpi.com/article/10.3390/ph16020308/s1, Table S1: Medications at the origin of the potential intoxication.

## References

[1] Eddleston M. (2000). Patterns and problems of deliberate self-poisoning in the developing world. QJM Int. J. Med..

[2] Smith S.W., Farmer B.M. (2015). Toxicology in the Service of Patient and Medication Safety: A Selected Glance at Past and Present Innovations. J. Med. Toxicol..

[3] Gokalp G. (2019). Evaluation of poisoning cases admitted to pediatric emergency department. Int. J. Pediatr. Adolesc. Med..

[4] Albano G.D., Malta G., La Spina C., Rifiorito A., Provenzano V., Triolo V., Vaiano F., Bertol E., Zerbo S., Argo A. (2022). Toxicological Findings of Self-Poisoning Suicidal Deaths: A Systematic Review by Countries. Toxics.

[5] Piccioni A., Cicchinelli S., Saviano L., Gilardi E., Zanza C., Brigida M., Tullo G., Volonnino G., Covino M., Franceschi F. (2020). Risk Management in First Aid for Acute Drug Intoxication. Int. J. Environ. Res. Public Health.

[6] European Monitoring Centre for Drugs and Drug Addiction (2019). Update from the EMCDDA Expert Network Drug-Related Deaths and Mortality in Europe.

[7] CIAV—Centro de Informação Antivenenos. https://www.inem.pt/category/servicos/centro-de-informacao-antivenenos/.

[8] European Monitoring Centre for Drugs and Drug Addiction (2021). Drug-Related Deaths and Mortality in Europe: Update from the EMCDDA Expert Network.

[9] Bian W., Zhu N., Han D., Gu F., Hu Y. (2021). Analysis of Influencing Factors of Acute Medication Poisoning in Adults in Emergency Department of Our Hospital from 2016 to 2019 and Observation of Curative Effect of Optimizing Emergency Procedures. Evid. Based Complement. Altern. Med..

[10] Gummin D.D., Mowry J.B., Beuhler M.C., Spyker D.A., Rivers L.J., Feldman R., Brown K., Nathaniel P.T.P., Bronstein A.C., Weber J.A. (2022). 2021 Annual Report of the National Poison Data System^©^ (NPDS) from America’s Poison Centers: 39th Annual Report. Clin. Toxicol..

[11] Hernández-Calle D., Martínez-Alés G., López-Cuadrado T. (2022). Suicidal and accidental drug poisoning mortality among older adults and working-age individuals in Spain between 2000 and 2018. BMC Geriatr..

[12] Martins S.S., Sampson L., Cerdá M., Galea S. (2015). Worldwide Prevalence and Trends in Unintentional Drug Overdose: A Systematic Review of the Literature. Am. J. Public Health.

[13] Cucinotta D., Vanelli M. (2020). WHO declares COVID-19 a pandemic. Acta Biomed..

[14] Guengerich F.P. (2011). Mechanisms of Drug Toxicity and Relevance to Pharmaceutical Development. Drug Metab. Pharm..

[15] Instituto Nacional de Estatística-Statistics Portugal Censos 2021. https://censos.ine.pt/xportal/xmain?xpgid=censos21_main&xpid=CENSOS21&xlang=pt.

[16] Serviço Nacional de Saúde (2021). Relatório Anual Atividades Específicas Desenvolvidas nos Centros de Orientação de Doentes Urgentes (CODU)-2020. https://www.inem.pt/wp-content/uploads/2021/04/Relatorio-de-Atividades-Especificas-CODU-2020.pdf.

[17] (2020). GDS Covid-19 Country Reports|Global Drug Survey. https://www.globaldrugsurvey.com/wp-content/themes/globaldrugsurvey/assets/GDS_COVID-19-GLOBAL_Interim_Report-2020.pdf.

[18] Hardon A., Hodgkin C., Fresle D. (2004). How to Investigate the Use of Medicines by Consumers.

[19] (2021). Centro de Informação Antivenenos. Centro de Informação Antivenenos-Dados Estatísticos 2021.

[20] Santana R., Rocha J., Sousa J., Soares P. (2021). A Procura de Serviços de Urgência/Emergência hospitlar: Tendênias durnte o primeiro mês de reposta à COVID-19. https://www.ensp.unl.pt/wp-content/uploads/2017/06/tendencia-de-resposta-dos-servicos-de-urg-emerg-covid-19.pdf.

[21] Entidade Reguladora da Saúde (2020). Informação de Monitorização-Impacto da pandemia COVID-19 no Sistema de Saúde—Período de Março a Junho de 2020, Porto. https://transparencia.sns.gov.pt.

[22] Ministério da Saúde SNS 24. https://www.sns24.gov.pt/.

[23] Gummin D.D., Mowry J.B., Beuhler M.C., Spyker D.A., Brooks D.E., Dibert K.W., Rivers L.J., Pham N.P.T., Ryan M.L. (2020). 2019 Annual Report of the American Association of Poison Control Centers’ National Poison Data System (NPDS): 37th Annual Report. Clin. Toxicol..

[24] Gummin D.D., Mowry J.B., Beuhler M.C., Spyker D.A., Bronstein A.C., Rivers L.J., Pham N.P.T., Weber J. (2021). 2020 Annual Report of the American Association of Poison Control Centers’ National Poison Data System (NPDS): 38th Annual Report. Clin. Toxicol..

[25] Kovess-Masfety V., Boyd A., Van de Velde S., De Graaf R., Vilagut G., Haro J.M., Florescu S., O’Neill S., Weinberg L., Alonso J. (2014). Are there gender differences in service use for mental disorders across countries in the European Union? Results from the EU-World Mental Health survey. J. Epidemiol. Commun. Health.

[26] Bertakis K.D., Azari R., Helms L.J., Callahan E.J., Robbins J.A. (2000). Gender differences in the utilization of health care services. J. Fam. Pract..

[27] Fundação Francisco Manuel dos Santos and PORDATA-Estatísticas Sobre Portugal e Europa Portugal: População Residente, Média Anual: Total e Por Grupo Etário|Pordata. https://www.pordata.pt/portugal/populacao+residente++media+anual+total+e+por+grupo+etario-10.

[28] Alves E.E., Brandão P., Magalhães T., Carvalho F., Dinis-Oliveira R.J. (2017). Fatal Intoxications in the North of Portugal: 12 Years of Retrospective Analysis. Curr. Drug Saf..

[29] Blaiss M.S., Deshazo R.D. (1988). Drug Allergy. Pediatr. Clin. N. Am..

[30] Sammons H.M., Choonara I. (2016). Learning Lessons from Adverse Drug Reactions in Children. Children.

[31] (2012). Safe Kids Worldwide. Safe Storage, Safe Dosing, Safe Kids A Report to the Nation on Safe Medication, Washington, D.C..

[32] Jerónimo M., Piñar S., Samos P., González A.M., Bellsolà M., Sabaté A., León J., Aliart X., Martín L.M., Aceña R. (2021). Intentos e ideas de suicidio durante la pandemia por COVID-19 en comparación con los años previos. Rev. de Psiquiatr. y Salud Ment..

[33] Kim J., de Jesus O. (2022). Medication Routes of Administration.

[34] Olsen S.J., Winn A.K., Budd A.P., Prill M.M., Steel J., Midgley C.M., Kniss K., Burns E., Rowe T., Foust A. (2021). Changes in Influenza and Other Respiratory Virus Activity During the COVID-19 Pandemic—United States, 2020–2021. MMWR Morb. Mortal. Wkly. Rep..

[35] Bellmann R., Joannidis M. (2017). Vergiftungen mit psychotropen Substanzen. Med. Klin. Intensiv. Notf..

[36] Furtado C., Cláudia F. (2013). Psicofármacos: Evolução do Consumo em Portugal Continental (2000-2012).

[37] Furtado C., Fernandes E. (2020). Evolução da pandemia em Portugal: Psicofármacos na última década em Portugal. Infarmed Notícias.

[38] Estrela M., Herdeiro M.T., Ferreira P.L., Roque F. (2020). The Use of Antidepressants, Anxiolytics, Sedatives and Hypnotics in Europe: Focusing on Mental Health Care in Portugal and Prescribing in Older Patients. Int. J. Environ. Res. Public Health.

[39] Ferreira R., Cunha B., Ferreira D.M., Devesa N., Pimentel J. (2016). Intoxicações Agudas num Serviço de Medicina Intensiva: Anos 2002 a 2014. Med. Interna-Rev. Soc. Port. Med. Interna.

[40] Prado D.M.L.D., Silvino V.O., Vieira E.G., Rosa B.V., e Silva A.S.V., dos Santos M.A.P. (2021). O Efeito da Máscara Cirúrgica de Proteção Respiratória nos Marcadores Fisiológicos de Desempenho Aeróbio em um Corredor Recreacional. Arq. Bras. de Cardiol..

[41] Paiva J.A., Lebre A., Silva M.G., Valente M., Pacheco P. Infeções e Resistências a Antimicrobianos-Relatório do Programa Prioritário PPCIRA 2021. https://www.dgs.pt/programa-nacional-de-controlo-da-infeccao/relatorios/infecoes-e-resistencias-aos-antimicrobianos-2021-relatorio-anual-do-programa-prioritario-pdf.aspx.

[42] Infarmed (2021). Medicamentos Não Sujeitos a Receita Médica (MNSRM)-Monitorização das Vendas fora das Farmácia (Jan-Dez 2020).

[43] United Nations and International Narcotics Control Board (2022). Psychotropic Substances-2021. Statistics for 2020. Assessments of Annual Medical and Scientific Requirements for 2022.

[44] Bouchette D., Akhondi H., Quick J. (2022). Zolpidem.

[45] Liu K., Chen Y., Wu D., Lin R., Wang Z., Pan L. (2020). Effects of progressive muscle relaxation on anxiety and sleep quality in patients with COVID-19. Complement. Ther. Clin. Prac..

[46] Rismanbaf A., Zarei S. (2020). Liver and Kidney Injuries in COVID-19 and Their Effects on Drug Therapy; a Letter to Editor. Arch. Acad. Emerg. Med..

[47] Santi P. Coronavirus: Pourquoi l’ibuprofène est Déconseillé. https://www.lemonde.fr/planete/article/2020/03/16/coronavirus-mise-en-garde-contre-l-ibuprofene_6033233_3244.html.

[48] Moore N., Carleton B., Blin P., Bosco-Levy P., Droz C. (2020). Does Ibuprofen Worsen COVID-19?. Drug Saf..

[49] Sridharan G.K., Kotagiri R., Chandiramani V.H., Mohan B.P., Vegunta R., Vegunta R., Rokkam V.R.P. (2020). COVID-19 and Avoiding Ibuprofen. How Good Is the Evidence?. Am. J. Ther..

[50] Poutoglidou F., Saitis A., Kouvelas D. (2021). Ibuprofen and COVID-19 disease: Separating the myths from facts. Expert Rev. Respir. Med..

[51] Infarmed (2022). Monitorização do Consumo de Medicamentos-Meio ambulatório. Janeiro-Dezembro 2021.

[52] Prontuário Terapêutico Infarmed-Autoridade Nacional do Medicamento e Produtos de Saúde. https://app10.infarmed.pt/prontuario/index.php.

