# Supplementary material for: Characterization of Potential Intoxications with Medicines in a Regional Setting
Source: Pharmaceuticals (Basel). 2023 Feb 16;16(2):308. doi: 10.3390/ph16020308 (PMC9965688; doi:10.3390/ph16020308)
Supplement: Supplementary file 1 [file pharmaceuticals-16-00308-s001.zip › pharmaceuticals-2164017-supplementary.pdf]

Table S1. Medications at the origin of the potential intoxication. Drugs are grouped according to their pharmaceutical classification and the respective number of times they were the cause of potential poisonings in 2019 and 2020 in Algarve based on calls to CIAV.

	2019	2020
**1. Anti-Infectious Drugs**		
**1.1 Antibacterials**		
**1.1.1. Penicillins**		
**1.1.1.2. Aminopenicillins**	13	2
**1.1.1.3. Isoxazolipenicillins**	1	-
**1.1.2. Cephalosporins**		
**1.1.2.1. 1st Generation Cephalosporin**	2	-
**1.1.2.2. 2nd Generation Cephalosporin**	1	3
**1.1.2.3. 3rd Generation Cephalosporin**	1	
**1.1.5. Combinations of Penicillins with Beta Lactamase Inhibitors**	9	10
**1.1.6. Chloramphenicol and Tetracyclines**	1	1
**1.1.8. Macrolides**	4	3
**1.1.10. Quinolones**	1	-
**1.1.11. Other Antimicrobials**	2	-
**1.1.12. Antituberculosis**	1	-
**1.3. Antivirals**		
**1.3.1. Antiretrovirals**		
**1.3.1.1. Protease Inhibitors**	2	1
**1.3.1.2. Integrase Inhibitors**	1	1
**1.3.1.3. Nucleoside reverse transcriptase inhibitors**	-	1
**1.4. Antiparasitic**		

1.4.2. Antimalarials	2	1
1.4.3. Other Antiparasitics	-	1
**2. Central Nervous System**		
2.3. Muscle Relaxants		
2.3.1. Central Action	13	1
2.5. Antiparkinsonics		
2.5.1. Anticholinergics	3	3
2.5.2. Dopaminergics		
Dopamine agonists	1	-
2.6. Antiepileptics and Anticonvulsants	124	62
2.7. Antiemetics and Antivertigo	8	2
2.8. Nonspecific Central Nervous System Stimulants	2	2
2.9. Psychoactive drugs		
2.9.1. Anxiolytics, Sedatives and Hypnotics		
Benzodiazepines	172	199
Plant Extracts	3	1
Zolpidem	25	6
Doxylamine	4	5
Melatonin	2	2
Cyclopyrrolones	1	-
2.9.2 Antipsychotics	86	109
2.9.3. Antidepressants		
Monoamine Oxidase Type A Inhibitors	2	-
Selective Serotonin Reuptake Inhibitors (SSRIs)	68	51

Serotonin and norepinephrine reuptake inhibitors (SNRIs)	14	13
Melatonin Receptor Agonists	5	4
Tricyclic and related products	34	47
Other	2	1
2.10. Analgesics and Antipyretics	67	78
2.12. Analgesics Narcotics	9	8
2.13. Other Central Nervous System Medications		
2.13.1. Medications Used in the Symptomatic Treatment of Altered Cognitive Function		
Drugs Used for the Symptomatic Treatment of Alzheimer's Dementia	4	1
Other	1	-
2.13.3. Drugs for the Treatment of Drug Dependence	2	4
3. Cardiovascular System		
3.2. Antiarrhythmics		
3.2.1. Sodium Channel Blockers (Class I)		
3.2.1. Class Ic (Flecainide type)	1	-
3.2.3. Repolarization Prolongers (Class III)	2	1
3.2.4. Calcium Entry Blockers (Class IV)	-	5
3.3. Sympathomimetics	-	1
3.4. Anti-hypertensives	1	-
3.4.1 Diuretics		
3.4.1.1. Thiazides and Analogs	1	3
3.4.1.2. Ansa Diuretics	1	7

3.4.1.3. Potassium-sparing diuretics	-	1
3.4.2. Renin-Angiotensin Axis Modifiers		
3.4.2.1. Angiotensin-converting enzyme (ACE) inhibitors	4	10
3.4.2.2. Angiotensin Receptor Antagonists (ARA)	8	6
3.4.3. Calcium channel blockers	4	6
3.4.4. Adrenergic Activity Depressants		
3.4.4.2. Beta blockers		
3.4.4.2.1. Cardiac Selective	17	7
3.4.4.2.2. Cardiac Non-selective	3	6
3.4.4.2.3. Beta and Alpha Blockers	5	-
3.4.4.3. Alpha 2 Central Agonists	-	1
Fixed-Dose Antihypertensive Combinations		
Fixed Associations of Renin-Angiotensin Axis Modulators and Calcium Entry Blockers		
Fixed Combinations of ARAs and Calcium Entry Blockers	-	2
Fixed combinations of ACE inhibitors and calcium entry blockers	-	2
Fixed (triple) combinations of Calcium Entry Blockers, Renin-Angiotensin Axis Modulators and Diuretics	1	1
Fixed-diuretic and Renin-Angiotensin axis modulator combinations		
Fixed Associations of ARAs and Diuretics	10	6

Fixed ACE inhibitors and Diuretic Associations	1	2
Fixed Associations of Diuretics and Modulators of the Renin-Angiotensin Axis and Calcium Entry Blockers		
Fixed combinations of ACE inhibitors and calcium entry blockers	2	6
3.5. Vasodilators		
3.5.1. Antiangina	1	2
3.5.2. Other vasodilators	1	1
3.6. Venotropics	3	-
3.7. Antidyslipidemics		
Statins	1	9
Fibrates	1	1
Fixed-drug Combinations Targeting Different Cardiovascular Risk Factors		
Statins + ACE inhibitors + Calcium Entry Blockers	1	-
4. Blood		
4.1. Antianemics		
Iron Deficiency Anemia		
4.1.1. Iron Composites		
Oral Iron Compounds	4	-
4.1.2. Drugs for the Treatment of Megaloblastic Anemias	2	-
4.3. Anticoagulants and Antithrombotics		
4.3.1. Anticoagulants		

4.3.1.2. Antivitamin K	1	1
4.3.1.3. Platelet Antiaggregants	6	6
4.3.1.4. Other Anticoagulants		
Direct oral anticoagulants (DOACs)	5	3
Other	1	-
**5. Respiratory Apparatus**		
**5.1. Antiasthmatics and Bronchodilators**		
5.1.1. Beta Adrenergic Agonists	5	2
5.1.2. Cholinergic Antagonists	1	1
5.1.3. Anti-Inflammatory		
5.1.3.2. Leukotriene Antagonists	8	1
5.1.4. Xanthines	3	-
5.1.7. Associations		
5.1.7.2. Combination of Bronchodilators with Corticoids	1	1
5.1.7.3. Combination of Bronchodilators with Expectorants	4	-
**5.2. Antitussives and Expectorants**		
5.2.1. Antitussives	5	-
5.2.2. Expectorants	7	2
5.2.3. Associations and Decongestant Medications	4	1
**6. Digestive Tract**		
**6.1. Drugs that act in the mouth and oropharynx**		
6.1.1. Topical Application	4	2
**6.2. Antacids and Anti Ulceratives**		
6.2.1. Antacids	-	1

6.2.2. Gastric Secretion Modifiers		
6.2.2.3. Proton Pump Inhibitors	16	5
6.3. Gastrointestinal Motility Modifiers		
6.3.1. Gastric Motility Modifiers or Prokinetics	5	2
6.3.2. Modifiers of Intestinal Motility		
6.3.2.1. Laxatives and Cathartics	2	-
6.3.2.1.2. Contact Laxatives	2	1
6.3.2.1.4. Osmotic Laxatives	1	-
6.3.2.2. Antidiarrheals		
6.3.2.2.1. Obstipants	1	-
6.3.2.2.3. Antiflatulents	1	1
6.4 Antispasmodics	3	1
6.6 Enzyme Supplements, Dairy Bacillus and Analogs	1	-
6.9 Drugs that work on the Liver and Bile ducts		
6.9.2. Drugs for Treatment of Biliary Kidney Disease	1	-
7. Genitourinary System		
Topical Medicines for Vagina Application		
7.1.2. Anti-Infectious	1	1
7.4. Other Drugs Used in Genitourinary Disorders		
7.4.2. Drugs Used in Problems of Urination		
7.4.2.1. Drugs Used in Urinary Retention	1	1
7.4.2.2. Medication for Urinary Incontinence	-	1
8. Hormones and Drugs Used in the Treatment of Endocrine Diseases		

8.1. Hypothalamic and Pituitary Hormones, their Analogues and Antagonists		
8.1.2. Posterior Lobe of the Pituitary	3	-
8.2. Corticosteroids		
8.2.2. Glucocorticoids	4	2
8.3. Thyroid Hormones and Anti Thyroid Hormones	4	7
8.4. Insulins, Antidiabetics and Glucagon		
8.4.1. Insulins		
8.4.1.1. Short Action	2	3
8.4.1.2. Intermediate Action	-	1
8.4.1.3. Long-Acting	1	2
8.4.2. Other Antidiabetics		
Biguanides	6	4
Sulfonylureas	1	-
Antidiabetic Associations	2	4
DPP4 Inhibitors - Gliptins	1	1
8.5. Sex Hormones		
8.5.1. Estrogens and Progestogens		
8.5.1.2. Contraceptives	12	9
8.5.1.3. Progestogens	6	2
9. Locomotor Apparatus		
9.1. Non Steroidal Anti-Inflammatory Drugs		
9.1.2. Acetic Acid Derivatives	6	6
9.1.3. Propionic Acid Derivatives	58	32
9.1.5. Indole and Indene Derivatives	5	2

9.1.6. Oxicams	-	2
9.1.7. Sulfanilamide Derivatives	4	3
9.1.9. Cox 2 Selective Inhibitors	-	2
9.1.10. Non Steroidal Anti-Inflammatories for Topical Use	1	1
9.3. Drugs Used to Treat Gout	4	-
9.5. Anti-Inflammatory Enzymes	2	3
9.6. Medicines that act on bone and on calcium metabolism		
9.6.2. Bisphosphonates	1	-
**10. Antiallergic Medicines**		
10.1. Anti-Histaminics		
10.1.1. Anti-Histaminic H1 Sedatives	9	4
10.1.2. Anti-Histaminic H1 Non-Sedatives	19	5
**11. Nutrition**		
11.1. Enteric Nutrition		
11.1.1. Oral Dietary Supplements	3	
11.3. Vitamins and Mineral Salts		
11.3.1. Vitamins		
11.3.1.1. Liposoluble Vitamins	8	4
11.3.1.3. Vitamin Associations	6	3
11.3.2. Mineral Salts		
11.3.2.1. Calcium, Magnesium and Phosphorus		
11.3.2.1.2. Magnesium	1	1
11.3.3. Associations	2	1

B-complex vitamins + Calcium	1	
**13. Drugs Used for Skin Affections**		
**13.1. Skin Application Anti-Infectives**		
**13.1.1. Antiseptics and Disinfectants**	12	7
**13.1.3. Antifungals**	1	-
**13.1.4. Antivirals**	-	1
**13.1.5. Antiparasitic**	2	3
**13.4. Acne and Rosacea Medicines**		
**13.4.2. Acne**		
**13.4.2.2. Systemic Action**	-	1
**13.5. Topical Corticosteroids**	1	-
**13.7. Healing aids**	1	-
**13.8. Other Drugs Used in Dermatology**		
**13.8.2. Antipruritics and Local Anesthetics**	1	-
**Anti-Histaminics**	10	-
**Other Associations**	1	-
**13.8.3. Preparations for Warts, Corns and Condylomata**	1	-
**14. Drugs Used in Otorhinolaryngological Affections**		
**14.1. Products for Nasal Application**		
**14.1.1. Decongestants**	3	6
**14.1.2. Corticosteroids**	-	1
**14.2. Products for Ear Application**	5	-
**15. Medicines Used for Ocular Disorders**		
**15.1. Topical Anti-Infectives**		

15.1.1. Antibacterial	4	-
15.2. Anti-Inflammatory		
15.2.3. Other Anti-inflammatory, Decongestant and Antiallergic drugs	2	-
15.4. Drugs Used to Treat Glaucoma		
15.4.3. Beta blockers	1	-
16. Antineoplastic and Immunomodulating Drugs	-	1
16.2. Hormones e Anti Hormones		
16.2.2. Anti-Hormones		
16.2.2.3. Aromatase Inhibitors	-	1
18. Vaccines and Immunoglobulins		
18.2. Bacterial Lysates		
Bacterial Lysate of H. influenzae, D. pneumoniae, K. pneumoniae, K. ozaenae, S. aureus, S. pyogenes, S. viridans, N. catarrhalis	-	1
Unknown Drug	-	6
Homeopathic	1	5
Other	3	5
